# Anesthetic Management of a Patient With Factor XIII Deficiency Undergoing Encephaloduroarteriosynangiosis (EDAS): A Case Report and Literature Review

**DOI:** 10.7759/cureus.106827

**Published:** 2026-04-10

**Authors:** Vikas Chauhan, Andrew Sexton

**Affiliations:** 1 Anesthesiology, University of Mississippi Medical Center, Jackson, USA; 2 Anesthesiology, Columbia University Irving Medical Center, New York, USA

**Keywords:** anesthesia, coagulopathy, corifact, edas, encephaloduroarteriosynangiosis, factor concentrate, factor xiii deficiency, literature review, moyamoya disease, perioperative care

## Abstract

Factor XIII (FXIII) deficiency is an extremely rare inherited coagulopathy that poses significant perioperative risks of delayed bleeding despite normal routine coagulation tests. Due to the routine normality of the standard coagulation screening tests in the affected patients, the diagnosis needs a high index of clinical suspicion. The implications for the perioperative environment are especially critical in neurosurgical procedures, in which uncontrolled hemorrhage can lead to either stroke, irreversible neurological damage, or death. We discuss the case of a 48-year-old woman with FXIII deficiency and Moyamoya disease who has undergone left-sided encephaloduroarteriosynangiosis (EDAS) surgery after receiving 40 units/kg of plasma-derived FXIII concentrate (Corifact®). Normotension and normocapnia were ensured intraoperatively, and estimated blood loss was 300 milliliters. The hemoglobin levels were stable. The patient was discharged on day 3 after the surgery without any hemorrhagic incidents. We also surveyed the literature on FXIII deficiency in the perioperative environment that has been published so far, especially on diagnostic methods, replacement, and the findings in the neurosurgical patients. Given the limited literature on FXIII deficiency in the context of EDAS and Moyamoya disease, this report contributes valuable clinical insight and supports the need for individualized, evidence-based perioperative strategies.

## Introduction

Factor XIII (FXIII), also known as the fibrin-stabilizing factor, is a transglutaminase that plays a crucial role in the final step of the coagulation pathway by crosslinking fibrin strands to stabilize clots and protect them from early fibrinolytic breakdown [1]. It circulates as a heterotetramer composed of two catalytic A subunits and two carrier B subunits (A2B2) [2]. Congenital FXIII deficiency occurs in approximately one in two million people worldwide, though higher prevalence has been noted in regions where consanguinity is more common [3].

The clinical spectrum of FXIII deficiency ranges from mild mucosal and superficial bleeding to life-threatening events such as spontaneous intracranial hemorrhage (ICH) and hemarthroses [4,5]. A hallmark of FXIII deficiency is delayed postoperative bleeding, characteristically occurring 24-48 hours after surgery despite seemingly effective intraoperative hemostasis, because mechanically weak clots are vulnerable to fibrinolytic breakdown. In severe congenital deficiency, umbilical stump bleeding during the neonatal period may serve as the initial clinical presentation, prompting diagnostic evaluation [3].

FXIII deficiency can be congenital or acquired. The congenital form is an autosomal recessive condition categorized as type I (quantitative deficiency of the A subunit), type II (qualitative deficiency of the A subunit), or, more rarely, type B (deficiency of the B subunit) [3]. Acquired FXIII deficiency may develop secondary to major trauma [6], autoimmune disease [7], hepatic cirrhosis [8], malignancy [9], and consumptive coagulopathies. Notably, routine screening tests - prothrombin time (PT), activated partial thromboplastin time (aPTT), thrombin time (TT), fibrinogen levels, and platelet count - are typically normal in FXIII-deficient patients, since FXIII acts downstream of fibrin formation. The first-line diagnostic test is a quantitative FXIII activity assay, followed by antigen testing to determine the type of deficiency [10]. FXIII deficiency should therefore be suspected in any patient presenting with unexplained postoperative bleeding and a normal coagulation profile.

Management of FXIII deficiency in the perioperative setting focuses on replacing the factor to achieve adequate hemostasis and minimize bleeding risk. Maintaining sufficient FXIII activity levels is essential to prevent both spontaneous and perioperative bleeding, although the optimal target levels may vary depending on surgical risks and individual patient factors [11]. Plasma-derived FXIII concentrate is considered the treatment of choice because of its targeted action, predictable pharmacokinetics, and long half-life, which allows sustained factor activity following a single dose [12,13]. Neurosurgical procedures represent a particularly high-risk setting in patients with FXIII deficiency. Reduced FXIII activity has been associated with an increased risk of postoperative hemorrhage following intracranial surgery, emphasizing the importance of preoperative optimization [14,15]. Encephaloduroarteriosynangiosis (EDAS) is an indirect revascularization procedure performed in patients with Moyamoya disease, a progressive condition characterized by stenosis of intracranial arteries and formation of fragile collateral vessels. This procedure aims to improve cerebral perfusion and has demonstrated favorable long-term outcomes in patients with steno-occlusive cerebrovascular disease [16]. Although FXIII deficiency is clinically significant, there is a paucity of literature regarding its perioperative management in neurosurgical procedures, with particularly limited data in patients undergoing revascularization procedures for Moyamoya disease. The co-occurrence of FXIII deficiency and Moyamoya disease in a single surgical patient represents a rare and previously underreported clinical scenario. The objectives of this report are to describe the anesthetic and perioperative management of such a patient undergoing EDAS surgery and to review the relevant literature on FXIII deficiency in the perioperative setting.

## Case presentation

The patient was a 48-year-old woman with a previous medical history of hypertension and hyperlipidemia who first presented to the hematology clinic before the removal of breast implants to be evaluated due to excessive bleeding after previous interventions. According to the patient, she had intra- and postoperative bleeding that complicated the course of multiple procedures, namely, the removal of a desmoid tumor of the chest wall, a benign lesion of the skin, cervical biopsy, and childbirth. Of note, she did not report having ever undergone any blood transfusion or surgical re-intervention in case of hemorrhage, implying a moderate phenotype.

Further evaluation for a possible bleeding disorder showed persistent normal PT, aPTT, TT, and platelet numbers, with increased fibrinogen and von Willebrand factor (vWF) activity. But the FXIII activity was reduced to 24%, and a second test confirmed 21% (reference range: 70-140%). These findings indicated a moderate FXIII deficiency. The perioperative laboratory profile is detailed in Table 1.

**Table 1:** Perioperative Laboratory Values * Confirmed on repeat testing. “Reference range” indicates values within the institutional reference range; exact values were not individually recorded for the routine screening panel. “-” indicates not measured. FXIII: factor XIII; PT: prothrombin time; aPTT: activated partial thromboplastin time; TT: thrombin time; vWF: von Willebrand factor; EBL: estimated blood loss; POD: postoperative day

Parameter	Preoperative	Intraoperative	POD 1	Reference Range
FXIII Activity (%)	24/21*	-	-	70-140
Hemoglobin (g/dL)	13.0	13.8	11.6	12.0-16.0
Platelet Count (×10^3^/μL)	182	-	174	150-400
PT (seconds)	12	-	12.5	11.0-13.5
aPTT (seconds)	31	-	32	25.0-36.0
TT (seconds)	15	-	17	14.0-19.0
Fibrinogen (mg/dL)	458	-	460	200-400
vWF Activity (%)	164	-	152	50-150
EBL (mL)	-	~300	-	-

The patient had subsequently suffered a mechanical fall and was observed to have a chronic infarct at the confluence of the posterior aspect of the right head of caudate and the body of caudate, and bilateral supraclinoid carotid artery occlusion, which is in line with Moyamoya disease. Cerebral angiography confirmed the diagnosis, and the patient was planned to undergo a left-sided EDAS. A clinical timeline of the patient’s course is presented in Table 2.

**Table 2:** Clinical Timeline of Patient’s Course FXIII: factor XIII; PT: prothrombin time; aPTT: activated partial thromboplastin time; TT: thrombin time; vWF: von Willebrand factor; ICA: internal carotid artery; EDAS: encephaloduroarteriosynangiosis; GA: general anesthesia; EBL: estimated blood loss; Hgb: hemoglobin; ICU: intensive care unit; POD: postoperative day

Timepoint	Event
Years prior	History of excessive intra- and postoperative bleeding following desmoid tumor excision, skin lesion removal, cervical biopsy, and childbirth; no transfusions or re-interventions required
Initial hematology referral	Evaluation for bleeding diathesis prior to planned breast implant removal; PT, aPTT, TT, and platelet aggregation normal; fibrinogen and vWF activity elevated; FXIII activity 24% (confirmed 21% on repeat)
Subsequent event	Mechanical fall; imaging reveals chronic caudate infarct and bilateral supraclinoid ICA occlusion; cerebral angiography confirms moyamoya disease diagnosis
Preoperative planning	Multidisciplinary consultation (hematology, neurosurgery, anesthesiology); decision to administer Corifact® (40 U/kg) preoperatively for left-sided EDAS
Day of surgery: Preoperative	FXIII concentrate (Corifact®) infused in the preoperative holding area without adverse effects
Day of surgery: Intraoperative	Radial arterial line placed; GA induced with propofol; video laryngoscopy intubation; strict normotension and normocapnia maintained; EBL ~300 mL; Hgb stable at 13.8 g/dL
Day of surgery: Postoperative	Uneventful extubation; transfer to neurosurgical ICU; stable hemodynamics and Hgb
POD 1	Hgb nadir 11.6 g/dL; no bleeding complications; no transfusion required
POD 3	Discharged home in good condition without hemorrhagic events

Given the nature of the bleeding disorder in the patient, a multidisciplinary decision was reached with consultation with the hematology team to administer plasma-derived FXIII concentrate (Corifact®) in the preoperative holding area to reduce the risk of excessive bleeding. FXIII was given in 40 units/kg intravenously to the patient without any adverse effects. On completion of the infusion, the patient was moved to the operating room, where the pre-induction radial arterial line was set up to monitor her hemodynamic status. Propofol induced general anesthesia, and video laryngoscopy endotracheal intubation was done successfully without any complication. Anesthetic care was focused on normotension and normocapnia maintenance of the procedure in order to reduce the ischemia and hemorrhage risks in the environment of moyamoya vasculopathy.

The surgical staff did not experience any excessive bleeding during surgery. A preoperative complete blood count revealed a hemoglobin level of 13.0 g/dL that was stable at an intraoperative level of 13.8 g/dL. The loss of blood incurred during the procedure was around 300 milliliters. The surgery was completed without any events, and the patient was extubated and transferred to the intensive care unit of neurosurgery. The postoperative course remained uneventful, with stable hemoglobin levels (nadir of 11.6 g/dL on day 1). There was no need for blood product transfusion. The patient was sent home on the third day after surgery in good condition.

## Discussion

Review of literature

Review Methodology

A literature search was conducted using PubMed, Google Scholar, and Scopus databases from inception through December 2024. Search terms included “factor XIII deficiency,” “FXIII deficiency,” “perioperative,” “surgery,” “neurosurgery,” “intracranial hemorrhage,” “factor replacement,” “corifact,” “fibrogammin,” and “encephaloduroarteriosynangiosis.” Boolean operators (AND, OR) were used to combine search terms. Inclusion criteria were English-language original studies, case reports, case series, and reviews addressing the epidemiology, diagnosis, perioperative management, or neurosurgical outcomes of FXIII deficiency. Exclusion criteria included non-English articles, editorials without original data, and studies exclusively addressing FXIII in non-surgical contexts (e.g., purely genetic or molecular characterization studies). Reference lists of identified articles were hand-searched for additional relevant sources. The findings from this non-systematic narrative review are summarized below.

Epidemiology and Pathophysiology of FXIII Deficiency

Congenital FXIII deficiency was first described by Duckert et al. in 1960 and remains among the rarest of the inherited bleeding disorders [17]. The estimated prevalence is approximately one in two million, though this figure may underestimate the true burden of disease given the limitations of standard coagulation screening in detecting FXIII deficiency. Higher prevalence rates have been reported in populations with increased rates of consanguinity, including parts of Iran, the Middle East, and South Asia [3,18]. The FXIII A subunit is synthesized primarily in bone marrow-derived cells, including monocytes and macrophages, while the B subunit is produced by hepatocytes [1,2]. Congenital deficiency most commonly involves the A subunit (type I or type II), with B subunit deficiency being exceedingly rare [3].

**Figure 1:**
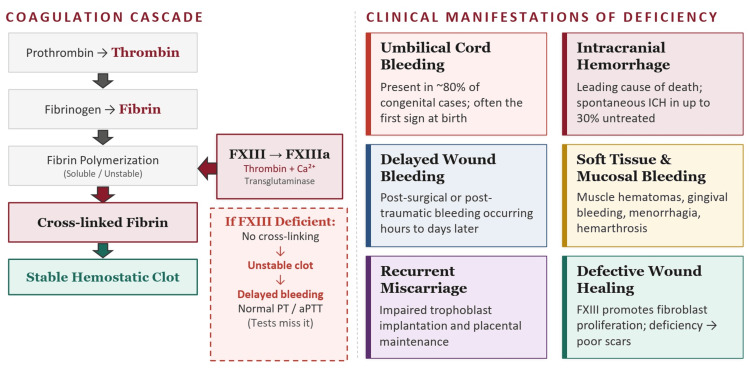
FXIII Deficiency: Mechanism and Clinical Effects The left panel illustrates the biochemical mechanism of FXIII activation and its role in fibrin crosslinking. The right panel depicts the clinical manifestations resulting from FXIII deficiency, including delayed postoperative bleeding, spontaneous ICH, and impaired wound healing. FXIII: factor XIII; Ca2+: calcium ions; PT: prothrombin time; aPTT: activated partial thromboplastin time; ICH: intracranial hemorrhage Source: Image created by the authors using Microsoft PowerPoint (Microsoft Corporation, Redmond, WA, USA).

Acquired FXIII deficiency, though also uncommon, can develop in a variety of clinical contexts. Hetz et al. demonstrated that FXIII deficiency is prevalent among multiply injured trauma patients, likely due to consumptive coagulopathy and hemodilution [6]. FXIII deficiency that is mediated by autoantibodies against the A subunit has been documented in conjunction with systemic lupus erythematosus (SLE) [7] and other autoimmune diseases. Hepatic cirrhosis may result in a fall in B subunit production and FXIII levels [8], and an acquired deficiency has been reported with regard to hematologic malignancy [9]. Figure 1 illustrates the FXIII deficiency mechanism and its related clinical effects.

Diagnostic Challenges

One of the inherent problems with the diagnosis of FXIII deficiency is that standard coagulation screening tests, such as PT, aPTT, TT, fibrinogen, and platelet count, are usually normal. The assays determine the upstream coagulation cascade via fibrin formation, rather than the cross-linking activity of FXIII, which occurs after fibrin polymerization [10]. A qualitative screening procedure is the clot solubility test, which is only moderately sensitive and has been largely replaced by quantitative tests of FXIII activity. A diagnostic algorithm that was suggested by Schroeder started with a functional FXIII activity test (ammonia release or isopeptidase-based), then the FXIII antigen test (A and B subunit) to determine the type of deficiency [10]. The level of FXIII below 70% of normal is considered deficient, although the risk of bleeding is related to the degree of deficiency, and the European Network of Rare Bleeding Disorders evidence indicated a close relationship between the level of FXIII activity and the degree of bleeding, with a proposed protective level of about 15 IU/dL to prevent significant spontaneous bleeds [11]. The levels of 24 and 21 percent FXIII activity in our case, on repeat testing, were also typical of a moderate deficiency and would place her at risk of significant hemorrhage, especially in the operating room.

FXIII Deficiency and Neurosurgical Outcomes

In a few studies, the quality of neurosurgical outcomes with respect to FXIII deficiency has been investigated. In a retrospective review of 1,264 patients who underwent intracranial surgery, Gerlach et al. identified an association between decreased FXIII activity and postoperative hemorrhage, noting that among 34 patients tested postoperatively for suspected coagulation abnormalities, 11 (32.3%) had suffered a major postoperative hemorrhage. The authors suggested that FXIII deficiency should be included in the diagnostic evaluation of unexplained post-neurosurgical bleeding [14]. The same group, in a later prospective study of 876 patients undergoing 910 neurosurgical surgeries, found that patients who experienced postoperative intracranial hematoma had much lower levels of preoperative and postoperative FXIII activity than those with no hematoma, and that 33.3% of those with postoperative hemorrhage had levels of FXIII activity below 60%, as compared with 7% in the non-hematoma group [15]. These findings indicated that reduced FXIII activity can be one of the independent predictors of postoperative neurosurgical bleeding.

One of the known complications of severe FXIII deficiency is spontaneous ICH [4]. Okumura et al. described an acquired FXIII deficiency secondary to SLE presenting with recurrent intracerebral hemorrhage, illustrating the diagnostic challenge posed by acquired forms [7]. In a comprehensive review of ICH among congenital bleeding disorders, Alavi et al. reported that approximately 30% of untreated patients with severe congenital FXIII deficiency experience spontaneous ICH, as the highest rate among all inherited coagulopathies [19]. These reports highlight the particular susceptibility of the central nervous system (CNS) to hemorrhagic events in FXIII-deficient patients and reiterate the necessity of preoperative factor correction before neurosurgical procedures.

Factor Replacement Strategies

Management of FXIII deficiency relies on replacement of the deficient factor. The fresh frozen plasma (FFP), cryoprecipitate, and FXIII concentrates are the available replacement options. Comparing the FXIII content of cryoprecipitate and the FXIII content of FFP, Caudill et al. showed that cryoprecipitate contains about two to three times the FXIII concentration of FFP per unit volume and is therefore more effective in terms of replacement of FXIII in the patient who is not able to tolerate the amount of fluid volume in FFP administration [20]. However, both FFP and cryoprecipitate carry risks of transfusion-associated adverse events, including allergic reactions, transfusion-related acute lung injury (TRALI), and pathogen transmission [21,22].

Plasma-derived FXIII concentrates, such as Corifact® (CSL Behring) and Fibrogammin® P (CSL Behring), as well as recombinant FXIII-A2 (Tretten®, Novo Nordisk), are all targeted replacements and provide benefits in the form of precision in dosing, lower volume of administration, and improvement in the safety profile over blood component therapy. Corifact® is indicated for use in the prophylactic routine and perioperative care of congenital FXIII deficiency. FXIII plasma half-life ranges between nine and 14 days, which offers long-term hemostatic protection during the normal perioperative and wound healing duration [2,12].

Janbain et al. reported on six patients with congenital FXIII deficiency who received preoperative Corifact®/Fibrogammin® P at doses ranging from 25 to 40 U/kg for major and minor surgical procedures [13]. Satisfactory intraoperative and postoperative hemostasis was achieved in the majority of cases, although one patient undergoing aortic valve replacement on cardiopulmonary bypass required additional intraoperative dosing due to lower-than-expected FXIII blood levels. This surgical case series demonstrated that preoperative administration of FXIII concentrate achieves rapid factor replacement and effective hemostasis across a range of procedures [13].

Additional evidence supports the efficacy of preoperative FXIII replacement across diverse surgical settings. Carneiro et al. reported that an FXIII-guided treatment algorithm significantly reduced blood transfusion requirements in patients undergoing burn surgery [23]. In the largest published case series on surgical outcomes in congenital FXIII deficiency, Naderi et al. reported on 162 patients undergoing minor, major, and obstetrical/gynecological surgeries at two major referral centers in Iran [12]. Plasma-derived FXIII concentrate (Fibrogammin® P) was administered at variable doses depending on the procedure type: 10 U/kg for minor procedures, 30 U/kg preoperatively and postoperatively for major surgery, and 50 U/kg preoperatively and postoperatively for neurosurgical procedures. Hemostasis was rated as excellent in the majority of cases, with only five postoperative complications (two bleeding events and three thromboses) across all procedures, resulting in an overall complication rate of 1.8% [18]. These data support preoperative FXIII concentrate administration as a safe and effective strategy for preventing hemorrhagic complications in deficient patients undergoing surgery.

Anesthetic Considerations for EDAS in FXIII-Deficient Patients

Anesthetic management of patients with Moyamoya disease undergoing EDAS demands meticulous attention to cerebral hemodynamics. The anesthesiologist must meticulously manage hemodynamics and ventilation throughout the surgery, with the goal of normotension and normocapnia. Normotension aims to preserve cerebral perfusion through fragile collateral networks while avoiding hypertension, which may precipitate hemorrhage from abnormal moyamoya vessels. Normocapnia is equally critical, as hypocapnia induces cerebral vasoconstriction, which can worsen ischemia in already compromised vascular territories, whereas hypercapnia causes vasodilation, which may increase the risk of hemorrhage. Adequate hydration and avoidance of hyperthermia are additional considerations for minimizing metabolic demand and reducing the risk of cerebral ischemia [16].

The presence of FXIII deficiency was an additional consideration in the anesthesia plan for our patient. Placement of a radial arterial line pre-induction allowed continuous monitoring of blood pressure and serial measurement of hemoglobin concentration via arterial blood gas analysis. With propofol-induced general anesthesia and video laryngoscopy use for intubation, a successful transition to general anesthesia was achieved while maintaining hemodynamic stability. The infusion of FXIII concentrate before going to the operating room was intended to achieve adequate factor levels before surgical incision, thereby creating a desired hemostatic state throughout the surgery. Also, viscoelastic tests such as rotational thromboelastometry (ROTEM) can be useful for real-time evaluation of clot quality in FXIII-deficient patients and might be considered a component of the perioperative monitoring plan [8].

Table 3 illustrates the key studies and their key findings.

**Table 3:** Summary of Key Studies on FXIII Deficiency in the Perioperative Setting Studies are listed in chronological order. This table contextualizes the current case within the existing evidence base and highlights the range of surgical settings in which FXIII replacement has been evaluated. FXIII: factor XIII; FFP: fresh frozen plasma; ICH: intracranial hemorrhage; CPB: cardiopulmonary bypass; N/A: not applicable; pts: patients; proc.: procedures

Author	Year	Study design	Sample size	Findings
Gerlach et al. [14]	2000	Retrospective cohort	1,264	FXIII deficiency identified in patients with post-neurosurgical hemorrhage; recommended FXIII screening in unexplained postoperative bleeding
Gerlach et al. [15]	2002	Prospective cohort	876 pts; 910 proc.	Decreased perioperative FXIII activity independently associated with increased risk of postoperative intracranial hematoma (33.3% vs. 7% with FXIII <60%)
Caudill et al. [20]	2009	Laboratory comparison	N/A	Cryoprecipitate contains 2-3x the FXIII concentration of FFP per unit volume
Janbain et al. [13]	2015	Retrospective case series	6	Preoperative Corifact®/Fibrogammin® P (25-40 U/kg) achieved satisfactory hemostasis in the majority of surgical cases; one CPB patient required additional dosing
Naderi et al. [18]	2017	Retrospective case series	162	Variable-dose FXIII concentrate (10-50 U/kg by procedure type) yielded excellent hemostasis; 1.8% postoperative complication rate
Alavi et al. [19]	2018	Narrative review	N/A	ICH occurs in ~30% of untreated severe congenital FXIII deficiency - the highest rate among all congenital bleeding disorders; replacement therapy is the mainstay of treatment
Carneiro et al. [23]	2018	Prospective interventional	Burn pts	FXIII-guided algorithm reduced blood transfusion requirements in burn surgery

Discussion

We present a successful perioperative management of FXIII deficiency in a high-risk neurosurgical case, and to our knowledge, reports describing the perioperative management of FXIII deficiency in patients undergoing EDAS for Moyamoya disease are extremely limited. The positive outcome of the case, with a small blood loss, stable hemoglobin level, and an uneventful postoperative course, aligned with the plan of preoperative administration of FXIII concentrate in combination with careful anesthetic management focused on hemodynamic stability.

There are several aspects of this case worth discussing. To start with, our patient’s diagnostic process can be characterized as a reflection of the even larger issue of FXIII deficiency. Although there was an evident clinical history of excessive postoperative bleeding in more than one surgical experience, the diagnosis could not be made until specific FXIII activity testing was done. The diagnostic blind spot in the normal outcomes of regular coagulation analyses in all the previous assessments highlights the need to identify FXIII deficiency in clinical practice. This case contributes to the literature, given the recommendation that FXIII activity be considered in the workup of patients with unexplained postoperative bleeding and otherwise normal coagulation profiles [10,14].

Second, the co-occurrence of FXIII deficiency and Moyamoya disease in our patient is of particular interest. While no direct pathophysiological link between the two conditions has been established, their coincidence increased the perioperative risk. Moyamoya disease predisposes to both ischemic and hemorrhagic stroke, and the fragility of collateral vascular networks complicates the management of any concurrent coagulopathy. This dual pathology demanded a delicate hemodynamic balancing act: sufficient perfusion pressure to sustain flow through tenuous collateral pathways had to be maintained without exceeding levels that could overwhelm the hemostatic capacity of an FXIII-deficient clot or rupture fragile moyamoya vessels.

Third, the choice of FXIII concentrate (Corifact®) over alternative replacement options (FFP or cryoprecipitate) in our case was guided by several factors: the ability to achieve precise dosing, the lower volume of administration (particularly advantageous in a neurosurgical patient where fluid overload may be detrimental), and the favorable safety profile relative to blood component therapy. Our management approach is consistent with current evidence supporting FXIII concentrate as first-line therapy and the need to achieve adequate perioperative FXIII activity levels to reduce bleeding risk [11,13,17]. The dose of 40 U/kg used in our patient is consistent with the manufacturer’s recommendation for perioperative management and aligns with the dosing range (25-40 U/kg) reported by Janbain et al. in their surgical case series using Corifact® [13]. It should be noted, however, that the dosing regimen employed here differs from the variable-dose protocol reported by Naderi et al., who used 50 U/kg specifically for neurosurgical procedures with Fibrogammin® P [18]. Both Corifact® and Fibrogammin® P are plasma-derived FXIII concentrates manufactured by CSL Behring. Given the long plasma half-life of FXIII (approximately 9-14 days) [2,17], a single preoperative dose should provide sufficient hemostatic coverage throughout the perioperative period and into early wound healing.

This report has certain limitations. This case report was prepared in accordance with the CAse REport (CARE) guidelines to ensure completeness and transparency of reporting. As a single-case study, generalizability is inherently limited. Postoperative FXIII activity levels were not measured, and such data would have provided additional insight into the concentrate's pharmacokinetics and the adequacy of replacement. Intraoperative viscoelastic testing (ROTEM) was not performed. Future research should aim to define optimal dosing, timing, and monitoring protocols for FXIII replacement in neurosurgical patients.

## Conclusions

FXIII deficiency is a clinically significant but under-recognized coagulopathy that evades standard screening tests and should be actively considered in patients with a history of unexplained postoperative bleeding. This report describes the successful anesthetic and perioperative management of a patient with the rare co-occurrence of FXIII deficiency and Moyamoya disease who underwent EDAS without hemorrhagic complications following preoperative FXIII concentrate administration. While this is a single case report and generalizability is inherently limited, the favorable outcome is consistent with the existing literature supporting the efficacy and safety of preoperative FXIII replacement in surgical patients. This case underscores the importance of targeted diagnostic investigation, individualized preoperative factor replacement, and multidisciplinary collaboration among anesthesiology, hematology, and surgery to optimize perioperative outcomes in patients with rare coagulopathies. Multicenter data and prospective studies are needed to further define optimal dosing, monitoring, and management protocols for FXIII-deficient patients undergoing neurosurgical procedures.
